# The Effect of a Dedicated Lung Mass Clinic on Lung Nodule Follow Up

**DOI:** 10.33552/aphe.2022.01.000524

**Published:** 2022-05-09

**Authors:** Avnee J Kumar, Dena H Tran, Barathi Sivasailam, Zain Nagaria, Jigar Patel, Avelino C Verceles, Janaki Deepak

**Affiliations:** 1 Division of Pulmonary and Critical Care Medicine, University of Michigan Medical Center, Ann Arbor, MI, USA; 2 Department of Medicine, University of Maryland Medical Center Midtown Campus, Baltimore, MD, USA; 3 Department of Medicine, University of Maryland Medical Center and Baltimore VA, Baltimore, MD, USA; 4 Division of Pulmonary and Critical Care Medicine, University of Maryland Medical Center and Baltimore VA, Baltimore, MD, USA; 5 Diagnostic Radiology and Nuclear Medicine, University of Maryland Medical Center Baltimore, MD, USA; 6 Imaging Services, VA Maryland Health Care System, Baltimore, MD, USA

## Abstract

**Introduction:**

With the increased use of computed tomography (CT) imaging, lung nodules are found yearly requiring tracking and guideline directed follow up imaging. We describe the structure of a clinic dedicated to lung nodule tracking, patient education and the outcomes of lung nodule follow up.

**Methods:**

Patient electronic medical record charts were reviewed for lung nodules requiring tracking to determine if a follow up study was ordered, completed by the patient, and completed in an appropriate time frame. Patients were grouped based on referral to pulmonary clinic, lung mass clinic, or no subspecialty clinic. 700 CT reports were extracted from the electronic medical record of which 350 (50%) had lung nodules reported on CT, and 111 (15.9%) were lung nodules that additionally recommended discrete follow up in the radiologist report at the Veterans Health Administration hospital in Baltimore. Of these 111 patients, 95% were male and 5% were female. The mean age of the population was 66.3 ± 7.7 years.

**Results and Discussion:**

Patients seen in the lung mass clinic had a statistically significant higher rate of the follow up study being ordered by the provider. The lung mass clinic also had a higher percentage of patients who completed the study and completed the study within the recommended time frame, however, this was not statistically significant.

**Conclusion:**

A dedicated lung mass clinic should be considered as a method of improving lung nodule tracking with the added benefit of patient education and multidisciplinary care.

## Introduction

Every year a multitude of lung nodules are detected on imaging either incidentally or during lung cancer screening [1]. In a study of eight Veteran’s Hospitals, approximately 60% of lung cancer screening computed tomography (CT) scans resulted in discovery of a nodule [2]. Once located, a nodule often requires further monitoring or workup as per set guidelines.

In the current process at the Baltimore Veterans Affairs (VA) Medical Center, physicians are notified of non-urgent pulmonary nodules via a paper-based reporting system. At the time of notification, the provider can refer the patient to a lung mass clinic, a pulmonary clinic, or a schedule to follow up CT scan independently. The lung mass clinic is designed to determine and complete appropriate assessment of lung nodules or masses.

Studies have shown that nodules are not followed up as recommended by guidelines, leading providers and institutions to re-evaluate their nodule tracking process [3]. Data on the utility of this type of specialty clinic is limited. We aimed to assess the rate of follow up for lung nodules at the Baltimore VA as stratified by clinic referral.

## Methods

CT scan reports containing the word ‘nodule’ were extracted from the Baltimore VA’s radiology report that were completed during the months of June and July of 2016. Reports with a lung nodule and a recommendation for follow up imaging by the radiologist were identified. Indication for CT was categorized into the following: symptoms suggestive of lung cancer, symptoms not likely related to lung cancer, lung cancer screening, follow up of a known pulmonary nodule, or other. CT report text was used to categorize the size, location, and description of the largest nodule. The electronic medical record (EMR) was reviewed to determine if follow up imaging was ordered by the provider and completed by the patient. The EMR was also used to determine if the patient was referred to or seen in the pulmonary clinic or the lung mass clinic. Those who were referred to neither pulmonary clinic or lung mass clinic were categorized as ‘no clinic’. Adequate follow up was defined as imaging completed 30 days after the recommended date or any time before the recommended date. This retrospective study was sanctioned by the Veteran’s Health Administration as a Quality Improvement project.

### Data Collection and Analysis

Demographics were collected on all subjects, including smoking status (yes or no), presence of history of Chronic Obstructive Pulmonary Disease (COPD) and nodule size. All data was expressed in mean ± standard deviation, or counts and percentage, unless otherwise specified. Means were compared using student’s t-test. Proportions were compared using Chi-square testing. Specifically, the proportion of patients who were not followed in clinic, versus followed in pulmonary clinic, versus followed in lung mass clinic but had a CT ordered were compared across clinic groups. Similarly, those with a CT completed and completed adequately were also compared across the same three clinic groups. A two tailed p-value of less than or equal to 0.05 indicated statistical significance.

## Results

At the Baltimore VA from June to July 2016, there were 700 CT reports with the word nodule (Table 1). Out of the 700 CT reports, 350 were lung nodules located on CT, and 111 were lung nodules that additionally recommended discrete follow up in the radiologist report. Of these 111 patients, 95% were male and 5% were female. The mean age of the population was 66.3 ± 7.7 years. There was no statistical significance in demographics between demographics including age, gender, race, or smoking status.

The reason for the initial CT scan was most often follow up of a known pulmonary nodule (Table 2). The mean time of recommended follow up by the radiologist was 6.7 ± 4.6 months. As shown in Table 1, 39 (35%) nodules were less than 5 millimeters (mm), 50 (45%) nodules were 5 to 8mm, 9 (8%) nodules were 9 to 10mm, 10 (9%) nodules were 11 to 20mm, 1 (0.9%) nodule was 21 to 30mm, and 2 (2%) nodules were greater than 30mm. Forty-two (37.8%) nodules were located in the upper lobe. Eighty-two (73.9%) were classified as solid without spiculation or lobulation, with the remaining 29 nodules split into solid with spiculation or lobulation, semi-solid, and pure ground glass (9.0%, 9.9%, 7.2% respectively).

Out of 111 patients, 88 (79.3%) had the follow up study ordered, 73 (65.8%) had the follow up study completed, and 54 (48.6%) completed adequate follow up. The 57 (51%) patients with inadequate follow up had a mean age of 67.4 ± 7.6 years. Thirty-one (54.4%) nodules were less than 5 millimeters (mm), 19 (33.3%) nodules were 5 to 8mm, 2 (3.5%) nodules were 9 to 10mm, 3 (5.3%) nodules were 11 to 20 mm, 0 (0.0%) nodules were 20-30mm, and 2 (3.5%) nodules were greater than 30mm. Twenty-six (45.6%) nodules were located in the upper lobe. Forty-three (75.4%) were classified as solid without spiculation or lobulation, with the remaining 14 nodules split into solid with spiculation or lobulation, semi-solid, and pure ground glass (10.5%, 7.0%, 7.0% respectively).

**Figure 1:**
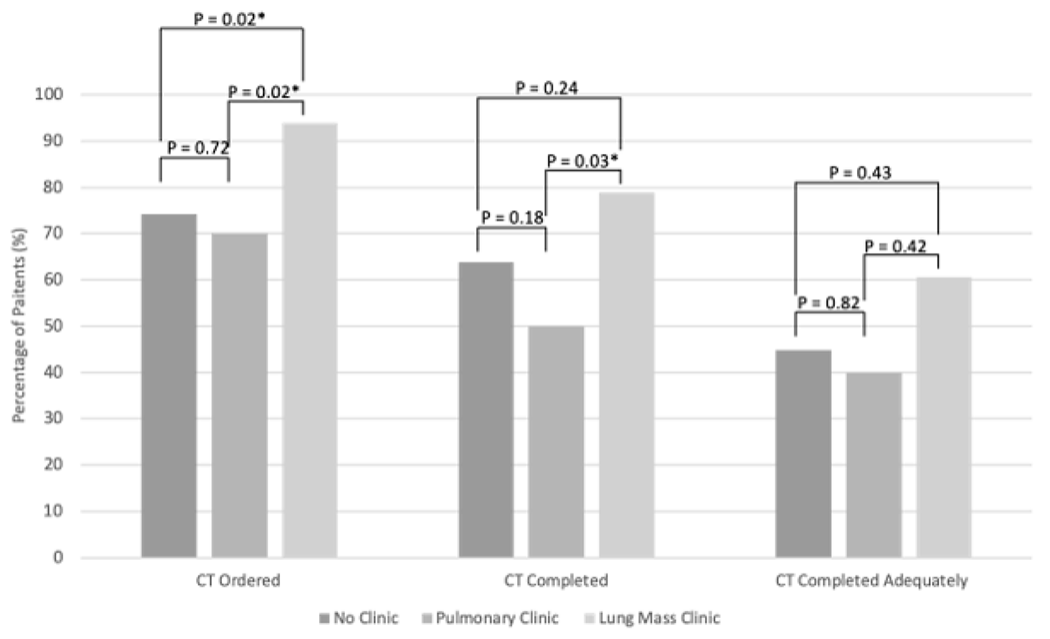
Percentage of patients who had the follow up study ordered, the follow up study completed, and the study completed within an adequate time frame as stratified by clinic referral.

Thirty-three (29.7%) patients were seen in lung mass clinic, 20 (18.0%) were seen in pulmonary clinic, and 58 (52.3%) were seen in neither clinic. Out of 33 patients referred to lung mass clinic, 31 (93.9%) had the follow up study ordered, 26(78.8%) had the follow up study completed, and 20 (60.6%) completed adequate follow up. Out of 20 patients referred to pulmonary clinic, 14 (70.0%) had the follow up study ordered, 10 (50.0%) had the follow up study completed, and 8 (40.0%) completed adequate follow up. Out of 58 patients referred to neither clinic, 43 (74.1%) had the follow up study ordered, 37 (63.8%) had the follow up study completed, and 26 (44.8%) completed adequate follow up (Table 3, Figure 1).

## Discussion

There was incomplete or delayed follow up of lung nodules detected on CT imaging at the Baltimore VA. Reasons for failure of tracking are multifactorial and include both system and patient factors. From a systems perspective, the process of nodule follow-up can be complicated. Incidental lung nodules are detected on medical imaging with increased frequency and tracking of high volumes of nodules can be difficult and resource consuming [1]. Once located, incidental nodules are generally followed up using guidelines created by the Fleischner Society, a multidisciplinary international group which primarily takes into account nodule size, morphology, location, multiplicity, and growth rate [4]. Guidelines attempt to simplify the other multitude of factors such as age, exposure, and location via high and low risk stratification. Furthermore, while the Fleischner criteria addresses the first nodule found on CT or one that is stable from prior CTs, the guidelines do not specifically address the patient with a previously normal CT and the development of a new nodule [4]. Furthermore, the Fleischner society guidelines do not apply to incidental nodules found in an immunocompromised host. Other aspects not clarified in the guidelines crucial to lung nodule management are attenuation, 3D evaluation, and solid size in a mixed nodule [5]. To add to the complexity, description and reporting of nodules can vary among radiologists, with interreader agreement decreasing with nodules below 8-10mm in size [6]. Notably, in a study from CHEST in 2017, when presented with a patient with a pulmonary nodule “physicians did not follow indicated guidelines when selecting the next test in 61% of cases”. However, physicians were more accurate in predicting malignancy than nodule prediction calculators [7].

From a patient perspective, lung nodules can be perplexing. A study from the Portland VA interviewed veterans with incidentally detected lung nodules. The study showed that patients did not understand the term ‘nodule’ or the follow up plan [8]. A longitudinal study published in JAMA from the same VA showed that even after two years of screening, veterans still felt underinformed [9]. Furthermore, patients have been shown to experience emotional distress when diagnosed with pulmonary nodules [10]. At the VA, using the My Healthy Vet patient portal, patients can now directly read radiologic reports without physician interpretation. While we support patients engaging in their own healthcare, reading about imaging abnormalities without proper context can potentially lead to increased patient confusion and anxiety.

The importance of tracking, despite its difficult nature, has led to increased efforts to streamline lung nodule tracking. In a VA-based study by Shelver, et al. [3], automated tracking systems resulted in significantly improved rates of adequate follow up [3]. An electronic discharge module implemented in the inpatient and ED setting showed improvement in follow up. Despite this, rates of follow up remained low at 27% [11]. A study evaluating the use of clinical pathway for unsuspected radiographic findings found that involving a pulmonary nurse specialist and pulmonary consultation team improved care [12]. Our data evaluates the potential of a lung mass clinic to help address the patient and system factors inherent in lung nodule tracking failure.

To our knowledge, there are two papers describing nodule clinics for patients with nodules discovered via screening [13, 14]. The first study, by Campo and Lennes, described the multidisciplinary care, navigation, and clinic setup of a nodule clinic. The second, a study by Gilbert, et al. [14], described the financial impact and potential revenue opportunity of a nurse practitioner run program on lung cancer screening, incidental pulmonary nodules, and tobacco-cessation services [14]. In our study, we present the data describing the tracking improvement in our VA-based lung mass clinic. In our data, veterans referred to lung mass clinic-with pulmonary consultation and a nurse specialist-had statistically significant improved rates of a follow up study being ordered as compared to those referred to pulmonary clinic or no subspecialty clinic. Additionally, the percentage of patients with CT scans completed and completed adequately were higher in lung mass clinic as compared to pulmonary clinic and no clinic, although this was not statically significant and therefore difficult to interpret. This lack of statistical significance may be as a result of a small sample size. Of note, the percentage of patients who completed a CT scan was also higher and statically significant between those referred to lung mass clinic versus pulmonary clinic. The reason for this is unclear and could be due to system and notification error.

A lung mass clinic can potentially address patient and system factors by providing optimal access to care, patient education with shared decision making, a multi-disciplinary team, a dedicated tracking system [15, 16], tobacco cessation, and up to date recommendations. Previous data has demonstrated patient distress related to lung nodule follow up, related to suboptimal patient understanding [8, 9]. A benefit of a lung mass clinic is a dedicated time to improve patient education on lung nodules and potential prognosis. As noted previously, a crucial component of lung nodule tracking is shared decision-making. Another evident benefit of a lung mass clinic is the use of a multidisciplinary team. Evaluation of pulmonary nodules is a growing field with potential novel imaging tests, biomarkers, and biopsy techniques offering improved diagnostic accuracy [17]. A lung mass clinic would have the specialty views to take ever changing tests into consideration. Furthermore, the VA clinic offers the potential of electronic consults (“e-consults”), which are electronic questions asked via Computerized Patient Record System (CPRS). This gives the clinic the opportunity to screen potential patients and offer guidance to clinicians with less time and scheduling efforts. Electronic consults allow for evaluation of patients without the constraints of scheduling [18].

Nodule clinics are also crucial given the increased importance and growing magnitude of lung cancer screening. The USPTF has a Grade B recommendation for lung cancer screening with low-dose CT scan in select adults aged 55 to 80 [19]. According to a 2017 study, 900,000 out of 6.7 million veteran men are estimated to meet criteria for annual lung cancer screening [20]. The arrival of this screening has been accompanied by the need for increased infrastructure. The American Thoracic Society and the American College of Chest Physicians released components necessary for high-quality lung cancer screening which, amongst other components, included structured reporting, smoking cessation, and lung nodule management algorithms [21]. Similar to when a nodule is found during screening, a nodule found incidentally requires shared decision making in order to determine appropriate follow up. A multidisciplinary team in a clinic setting could do this in a streamlined way.

## Conclusion

With incidental lung nodule discovery and increasingly used screening guidelines, optimizing methods to track lung nodules is necessary. Automated tracking systems can potentially address this need. Our data demonstrated referral to a dedicated lung mass clinic improved rate of follow up and should be considered as part of lung cancer screening programs.

## Figures and Tables

**Table 1:** Demographics of Subjects According to Clinic Type.

	All Patients (n=111)	No Clinic (n = 58)	Pulmonary Clinic (n = 20)	Lung Mass Clinic (n=33)	p-value
Age, years (mean ± SD)	66.3 ± 7.7	66 ± 6.9	69.4 ± 6.4	64.9 ± 9.3	0.11
Gender					0.89
Male	106 (95%)	55 (95%)	19 (95%)	32 (97%)	
Female	5 (5%)	3 (5%)	1 (5%)	1 (3%)	
Race					0.1
African American	48 (43%)	31 (53%)	9 (45%)	8 (24%)	
Caucasian	60 (54%)	26 (45%)	10 (50%)	24 (73%)	
Other	3 (3%)	1 (2%)	1 (5%)	1 (3%)	
Smoking Status					0.13
Yes	49 (44%)	27 (46%)	6 (30%)	16 (48%)	
No	11 (10%)	8 (14%)	0 (0%)	3 (9%)	
Prior	51 (46%)	23 (40%)	14 (70%)	14 (42%)	
COPD					0.002
Yes	40 (36%)	15 (26%)	14 (70%)	11 (33%)	
No	71 (64%)	43 (74%)	6 (30%)	22 (67%)	
Nodule Size					
<5mm	39 (35%)	20 (34%)	13 (65%)	6 (18%)	0.003
5-8mm	50 (45%)	28 (48%)	4 (20%)	18 (55%)	0.04
9-10mm	9 (8%)	6 (10%)	1 (5%)	2 (6%)	0.66
11-20mm	10 (9%)	3 (5%)	2 (10%)	5 (15%)	0.27
21-30mm	1 (0.9%)	1 (2%)	0 (0%)	0 (0%)	0.63
>30mm	2 (2%)	0 (0%)	0 (0%)	2 (6%)	0.09

Data presented as mean ± standard deviation (SD) or sample size, n (%)

**Table 2:** CT Scan Indication. Primary reason for ordering initial CT scan.

Indication	(n=111) No. (%)
Symptoms suggestive of lung cancer	6 (5.4%)
Symptoms not likely related to the nodule	24 (21.6%)
Lung cancer screening	19 (17.1%)
Follow-up of a known pulmonary nodule	46 (41.4%)
Other	16 (14.4%)

**Table 3:** CT Scan Performed According to Type of Clinic.

	All Patients (n=111)	No Clinic (n=58)	Pulmonary Clinic (n=20)	Lung Mass Clinic (n=33)	p-value
CT Ordered	88 (79.3%)	43 (74.1%)	14 (70%)	31 (93.9%)	0.04
CT Completed	73 (65.8%)	37 (63.8%)	10 (50%)	26 (78.8%)	0.1
CT Completed Adequately	54 (48.6%)	26 (44.8%)	8 (40%)	20 (60.6%)	0.65
